# Isothermal heat flow calorimetry for one-step determination of polymerization heat and rate of poly(acrylic acid) at varying pH

**DOI:** 10.1039/d5ra09826b

**Published:** 2026-02-18

**Authors:** Sebastian Remke, Gaurav Sant, Torben Gädt

**Affiliations:** a Chair for the Chemistry of Construction Materials, TUM School of Natural Sciences, Technical University of Munich Lichtenbergstraße 4, 85748 Garching Germany torben.gaedt[at]tum.de; b Laboratory for the Chemistry of Construction Materials (LC^2^), Department of Civil and Environmental Engineering, University of California Los Angeles Los Angeles CA 90095 USA; c Institute for Carbon Management (ICM), University of California Los Angeles Los Angeles CA 90095 USA

## Abstract

The radical polymerization of olefinic monomers in solution is an exothermic process. Despite the widespread use of acrylic acid, the pH dependence of its polymerization heat has not been reported. In this study, we employ time-corrected isothermal heat-flow calorimetry to simultaneously determine the heat and the rate of polymerization of acrylic acid at different degrees of neutralization ranging from 0% to 125%. We find a heat of (72.5 ± 0.6) kJ mol^−1^ under acidic conditions and a maximum value of (75.3 ± 2.3) kJ mol^−1^ at a neutralization degree of 75%. Additionally, we demonstrate that the time correction of the experimental calorimetry data enables the determination of the polymerization rate of acrylic acid at different degrees of neutralization, yielding values comparable to those reported in previous studies. Therefore, the calorimetric experiment reported here allows the determination of the heat of polymerization and the rate of polymerization of acrylic acid in a single experiment.

## Abstract 2

Isothermal calorimetry, incorporating a time-correction method, was employed to measure the pH-dependent polymerization heat and polymerization rate of acrylic acid. Subsequent size-exclusion chromatography provided molar mass and polydispersity data.

## Graphical abstract

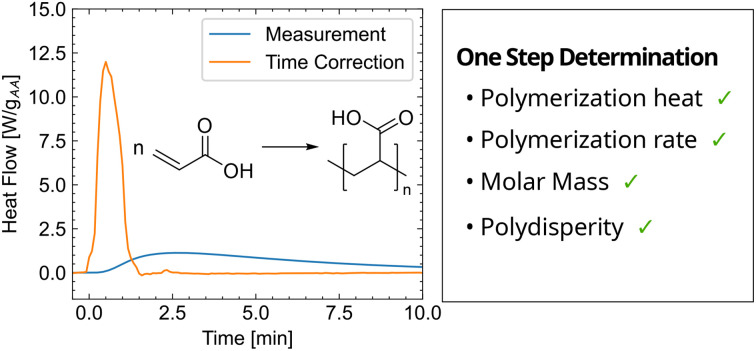

## 1 Introduction

Acrylic acid is used as a precursor for the production of superabsorbers, acrylate-based emulsion polymers, and solution polymers.^1^ Polyacrylic acids have many applications, such as dispersants, thickeners, hydrogels, or bioactive polymers.^2–4^ The influence of the pH, the ionic strength, and monomer concentration on the kinetics of acrylic acid polymerization has been studied extensively.^2,5–11^ Typical techniques for measuring kinetic data include infrared spectroscopy^2^ and dilatometry,^5,6^ nuclear magnetic resonance spectroscopy (NMR),^7^ Raman spectroscopy,^9^ differential scanning calorimetry (DSC)^10^ or light scattering.^11^

The heat of polymerization of acrylates is a comparatively less-studied parameter. Nevertheless, the polymerization heat is a valuable experimental parameter for determining acrylic acid conversion from the experimentally measured heat.^8,10,12–14^ The reported heat values for the polymerization of acrylic acid vary significantly from 59.0 kJ mol^−1^ to 77.4 kJ mol^−1^.^8,15–19^ This is problematic since the polymerization heat is needed for thermal hazard evaluation.^19,20^ Solvent effects are one of the reasons for this wide range of reported heat values.^8^ Another reason is the difficulty in measuring the heat of polymerization. To calculate the polymerization heat, the reaction heat and the polymer yield must be determined precisely. The determination of the polymer yield is often conducted by gravimetrical means^15^ or by titration.^16,18^

The heat measurement is either conducted by a custom-built calorimeter,^15,16^ differential scanning calorimetry^19^ or by isothermal calvet calorimetry.^8,18^ Although acrylic acid is widely employed in free-radical polymerization, the influence of pH on the reaction enthalpy and the associated rate has not been quantified systematically. This study aims to determine both parameters as functions of the pH value and to obtain them simultaneously within a single experiment.

Building upon our experience with isothermal heat flow calorimetry in cement chemistry,^21–27^ we apply this technique to the solution polymerization of acrylic acid. By using *in situ* mixing and applying a dynamic time correction (Tian equation),^23–25,27–29^ we demonstrate that the polymerization rate and enthalpy can be determined simultaneously. This approach allows for the characterization of thermokinetic properties across varying degrees of neutralization.

## 2 Experimental

### 2.1 Chemicals

Acrylic acid (AA, 98%, stabilized with 4-methoxyphenol) and sodium persulfate (98%) were obtained from Acros Organics, trisodium nitrilotriacetate (98%) and 4-methoxyphenol from Sigma Aldrich, iron(ii) sulfate heptahydrate (99%) and acetone (99.5%) from Chemsolute, disodium EDTA dihydrate (99%; EDTA = ethylenediaminetetraacetic acid) and sodium hydroxymethanesulfinate (98%) from Roth, sodium hydroxide (99%) from Grüssing GmbH, *tert*-butyl hydroperoxide (70% aqueous solution) from Alfa Aesar, and formic acid (99.9%, CHROMANORM) as well as acetonitrile (99.9%, CHROMANORM) from VWR.

Ultrapure water (NANOpure Diamond, Barnstead) was used for all experiments. Acrylic acid was distilled and diluted to a 20% aqueous solution containing 200 ppm 4-methoxyphenol as an inhibitor before usage.

### 2.2 Determination of monomer conversion by high-performance liquid chromatography (HPLC)

High-performance liquid chromatography (HPLC) was performed on a Thermo Fisher Ultimate 3000 series (Pump: LPG 3400SD, Autosampler: WPS 3000SL ANALITICAL, UV detector: DAD 3000) with an Agilent Polaris 5 NH_2_ (3 mm × 140 mm, particle size: 5 µm) column and UV detection (*λ* = 215 and 254 nm) at 20 °C. Eluent A was ultrapure water provided by a Merck Millipore Direct-Q 3UV containing 0.1% formic acid. Eluent B was acetonitrile containing 0.1% formic acid.

Samples were prepared by diluting 200 µL of polymer solution to 1 mL or by taking 1 mL sample solution (for acrylic acid conversions over 80%). Polyacrylic acid was precipitated by adding 0.5 mL sodium hydroxide and 0.5 mL acetone. The precipitate was removed by a syringe filter (0.22 µm). The resulting solution was then measured to obtain the acrylic acid conversion.

The HPLC system was operated at a flow rate of 1 mL min^−1^. The run employed a linear gradient from 5% to 100% eluent B over 30 min, followed by an isocratic hold at 100% eluent B for 5 min. Eluent B was then decreased to 50% over 3 min and held at that ratio for 4 min. With this method, acrylic acid had a retention time of 1.87 min and was detected at 254 nm. Calibration was done in the range of 0.5 mg mL^−1^ to 4 mg mL^−1^.

### 2.3 Molecular weight determination by gel permeation chromatography

Gel permeation chromatography (GPC) measurements were performed on a SECcurity^2^ GPC-System (PSS Polymer Standards Service GmbH). The system consists of an Agilent 1260 Infinity II Isocratic Pump equipped with an upstream degasser, an Agilent 1260 Infinity II vial sampler, a temperature-controlled column compartment at 35 °C containing a SUPREMA pre-column (8 mm × 50 mm; particle size: 5 µm) and three main columns (8 mm × 300 mm; particle size: 5 µm; pore sizes: 30 Å, 1000 Å, 1000 Å). An Agilent 1260 Infinity II refraction index detector and a PSS SLD7100 multi-angle static light scattering detector were used for detection. All measuring angles (35°, 75°, 90°, 105°, 130°, and 145°) were used for the calculation of the molecular weights from the light scattering detector. The eluent is an aqueous buffer solution of 0.07 mol L^−1^ Na_2_HPO_4_ and 200 mg L^−1^ NaN_3_. A flow rate of 1 mL min^−1^ was applied. The light scattering detector was calibrated with a pullulan standard (molecular weight = 110 000 g mol^−1^). A literature value of 0.165 was taken as the dn/dc coefficient.^4^ All reported molecular weights were obtained from the light scattering detector data. The reference standards were obtained from PSS Polymer Standards Service GmbH.

### 2.4 Isothermal heat flow calorimetry

#### 2.4.1 Mixing reaction components inside the calorimeter using an *in situ* cell

**Fig. 1:**
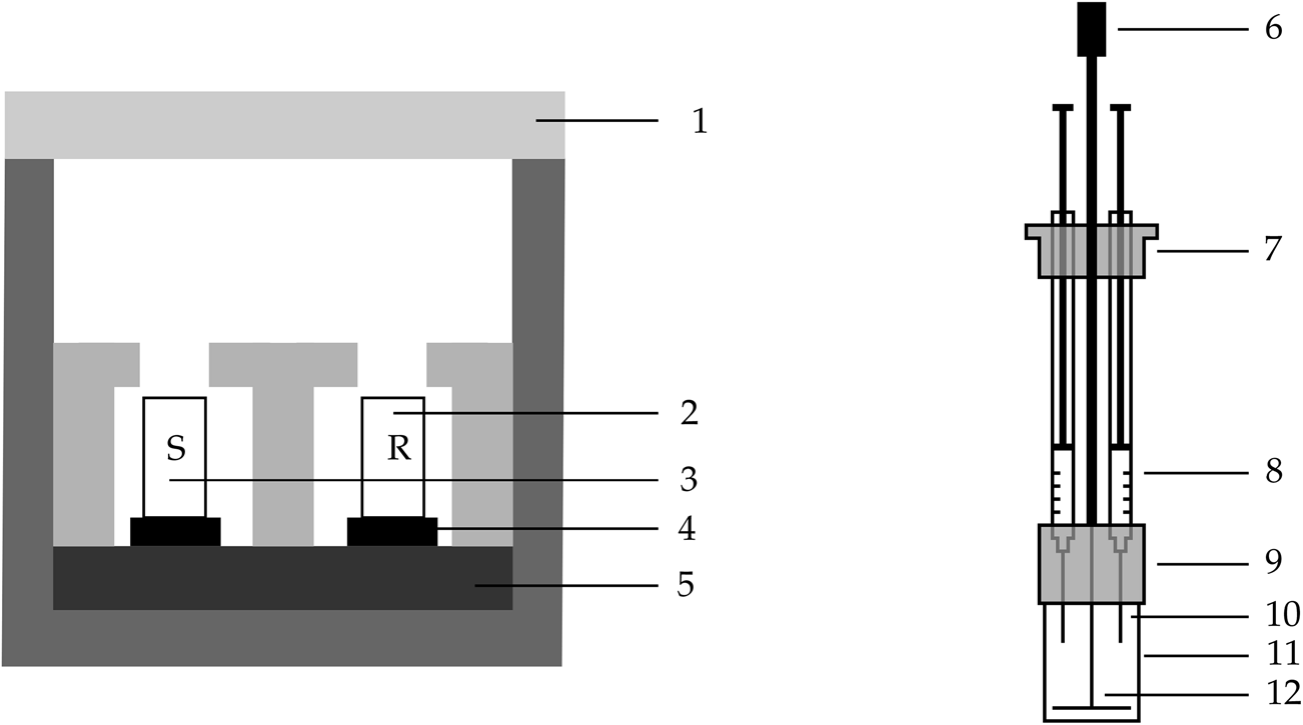
General schematic of an isothermal calorimeter (left) and the *in situ* mixer (right): (1) insulation lid, (2) reference, (3) sample, (4) heat flow sensor, (5) heat sink, (6) mount for electrical motor, (7) cap to close the headspace of the calorimeter, (8) syringes containing liquid, (9) cap to close the calorimeter cell, (10) cannula, (11) sample vial and (12) mixing paddle.

Heat flow measurements were conducted on an eight-channel isothermal calorimeter (TAM Air, TA Instruments, USA) at 20 °C. Custom mixers equipped with a T-shape paddle and four syringes were used (see Fig. 1 for the sketch); for most experiments, only three of the four syringes were utilized. These allow solutions to be mixed inside the calorimeter. The sample vial has a volume of approximately 20 mL and is composed of a polyamide/graphite composite. An external electrical motor is attached to the stirrer shaft (see item 6 in Fig. 1). The stirrer speed is set to 600 rpm for 90 s to ensure complete mixing while minimizing viscous heating. Throughout the experiments, the maximum temperature deviation of the heat sink was 0.002 K.

A calorimetry experiment was conducted by weighing water, acrylic acid, and sodium hydroxide (1 mol L^−1^) into the sample vial. Syringes were filled with the redox starter system (see Table 1). The peroxide, sodium hydroxymethanesulfinate, and iron(ii) solution were weighed into individual syringes. The total sample weight was 4 g, with an acrylic acid content of 2 weight percent; the water content was adjusted accordingly. After the mixer was fully assembled, including the mounted syringes, it was inserted into the calorimeter, together with a reference sample of water with a matching heat capacity (see Fig. 1), and equilibrated overnight. A baseline determination was conducted shortly before the measurement. The measurement was then started under stirring (600 rpm); after 30 s, the solution in the syringes was added to the sample vial, and stirring continued for 60 s. Experiments were run for 3 h, and the heat flow data were normalized to the acrylic acid content.

**Table 1:** Composition of polymerization reactions. The amounts are given in mol percent for the different initiator systems

	TBHP 2%	TBHP 5%	NaPS 5%	NaPS 10%
Acrylic acid	100	100	100	100
TBHP^a^	2	5	—	—
SFS^b^	2.2	5.5	—	—
FeSO_4_ × 7H_2_O	0.02	0.05	0.05	0.10
EDTA^c^	0.02	0.05	0.05	0.10
NaPS^d^	—	—	5	10

a *Tert*-butyl hydroperoxide.

b Sodium hydroxymethanesulfinate.

c Ethylenediamine tetraacetic acid tetrasodium salt.

d Sodium persulfate.

For each polymerization system in Table 1, we performed four heat-flow experiments: two polymerization runs (as detailed above), one reaction designed to record the heat of the redox reaction in which acrylic acid was replaced by propionic acid (referred to as “redox run”), and one control run in which both acrylic acid and the peroxide initiator were omitted. Propionic acid was selected as the non-reactive saturated analogue to acrylic acid to mimic the heat capacity and solvation environment of the monomer without participating in the radical chain reaction. By selective subtraction of the redox and control runs, we isolated (a) the net polymerization heat, (b) the heat of the redox side reaction, and (c) the viscous energy dissipation due to the stirrer.

#### 2.4.2 Time correction of the calorimetry data

To correct for the thermal delay in the calorimeter, a dynamic correction adapted from Evju *et al.* was used (eqn (2)).^23,27^ Where *U* is the measured signal in V, *ε* is the calibration coefficient from the calorimeter in W V^−1^ (usually already applied at the calorimeter), ε* is the individual correction factor, and *τ*_1_ and *τ*_2_ are time constants which affect the slope of a signal.

**Fig. 2:**
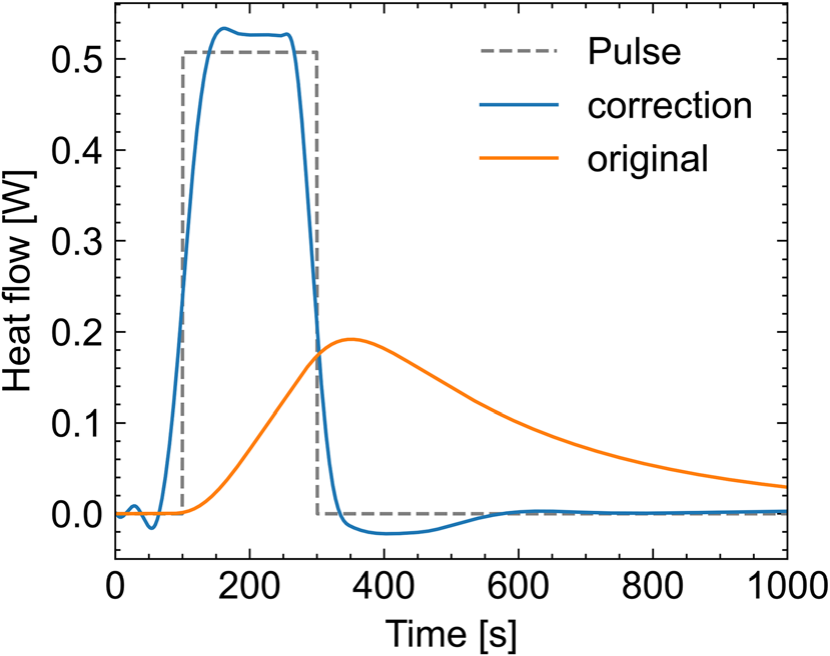
The applied heat signal by the resistor (pulse, dashed curve), the measured signal by the calorimeter (original, orange curve), and the result data (correction, blue curve) for the use of eqn (2) optimised to match the measured (original data) to the pulse fitted data.

These factors are determined using a calibration experiment using a 1774 Ω resistor inside the sample cell. The resistor was glued to the stirring paddle of the cell (approximately in the middle of the solution, not touching the bottom of the sample vial). The sample vial was filled with the same amount of water as used for the reference sample. After the assembly of the mixer and equilibration overnight, a heat signal was generated by applying 30 V for 200 s at the resistor (voltage was applied through small wires going through the syringe holes, the resistance of the resistor is several magnitudes higher than the wires, so the resistance of the wire was ignored). This generated a heat signal in a square shape (pulse in Fig. 2) and measured signal (original in Fig. 2). The fraction between calculated heat (by the resistor, *Q*_calc_) and measured heat (by the calorimeter, *Q*_meas_) is the individual correction factor ε* with a value of 1.044 for our system.

1 *Q*_calc_ = ε* × *Q*_meas_

In the next step, eqn (2) was used to fit the measured data (original) to the applied heat signal (pulse). This resulted in values of 316.43 s for *τ*_1_ and 57.91 s for *τ*_2_. Eqn (1) was used for all measurements. Eqn (2) with *τ*_1_ and *τ*_2_ was only used for kinetic considerations (Section 3.6).2
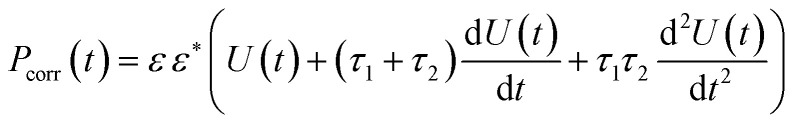


After this data treatment, the total evolved heat was taken after 3 h to ensure full capture of the heat event. The initial polymerization rate at 5% acrylic acid conversion.

## 3 Results and discussion

### 3.1 Identification of a redox system as initiator

The objective of this study is to apply heat flow calorimetry to determine both the heat of polymerization and the polymerization kinetics of acrylic acid at different pH values. First, we need to establish a reliable initiation method that effectively starts polymerization at room temperature. Common methods for initiating radical polymerization include thermal decomposition of labile initiators, UV-light-triggered radical generation, and redox-mediated initiator radical formation. Heat-triggered initiation is not ideal for the isothermal calorimeter setup because the *in situ* mixing cells are not sealed perfectly due to the mixer shaft protruding through the calorimeter top. UV-light-triggered polymerization would avoid this problem but would also require significant modification of available commercial calorimeters (including a lamp in the cell). Therefore, we choose redox initiation as a well-studied initiation method at ambient temperatures.^14,30–35^

The calorimetry cell, containing the vial with the monomer solution, was thermally equilibrated overnight with the reactant solutions in the syringes in the calorimeter at 20 °C. The relatively long resting time at 20 °C required a stable oxidant, which is why *tert*-butyl hydroperoxide (TBHP) was chosen as radical source. Sodium formaldehyde sulfoxylate (SFS) was selected as the corresponding reductant as a matching redox partner for the decomposition reaction of *tert*-butyl hydroperoxide.^33^

Initial tests with TBHP/SFS showed variable induction times. The addition of iron(ii) sulfate as a catalyst, stabilized with EDTA,^33^ eliminated this variance (see SI for details).

**Fig. 3:**
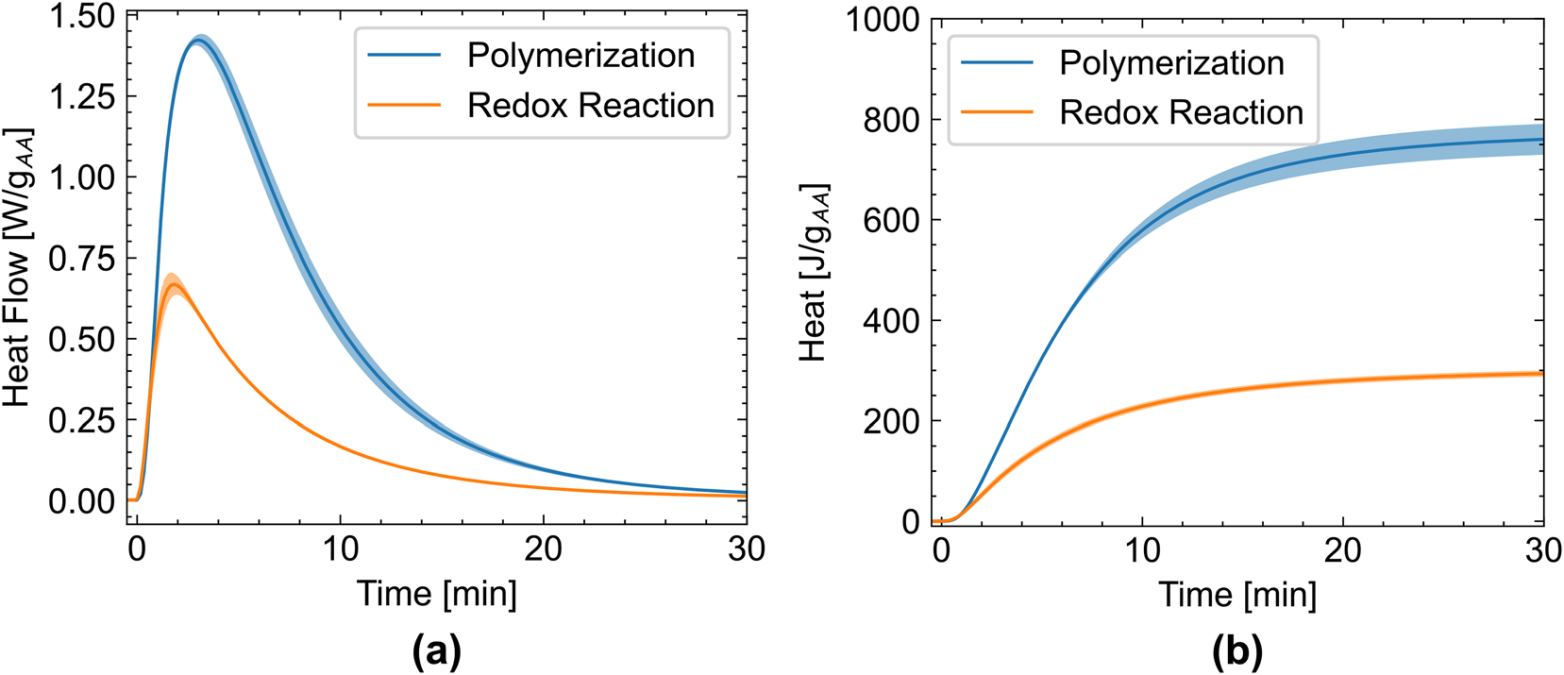
(a) Comparison of the heat flow between the polymerization reaction and the redox reaction, normalized to the acrylic acid content. (b) Comparison of the cumulative heat. Note that propionic acid instead of acrylic acid was used for the determination of the heat of the redox reaction. The mean value of 4 measurements is shown, and the standard deviation is depicted as the shaded area.

The combination of TBHP/SFS and iron(ii)–EDTA solution resulted in a well-timed and heat-consistent redox reaction (Table 1 and Fig. 3). This results in highly reproducible polymerization kinetics and yields polyacrylic acid with a molar mass of (19.7 ± 0.9) kg mol^−1^ and a PDI of (1.75 ± 0.05). The redox system, comprising TBHP/SFS/Fe(ii) sulfate/EDTA, in a molar ratio 2/2.2/0.02/0.02 to 100 parts of acrylic acid (Table 1), was used for all further polymerization experiments.

### 3.2 Determination of the polymerization enthalpies at different pH

**Fig. 4:**
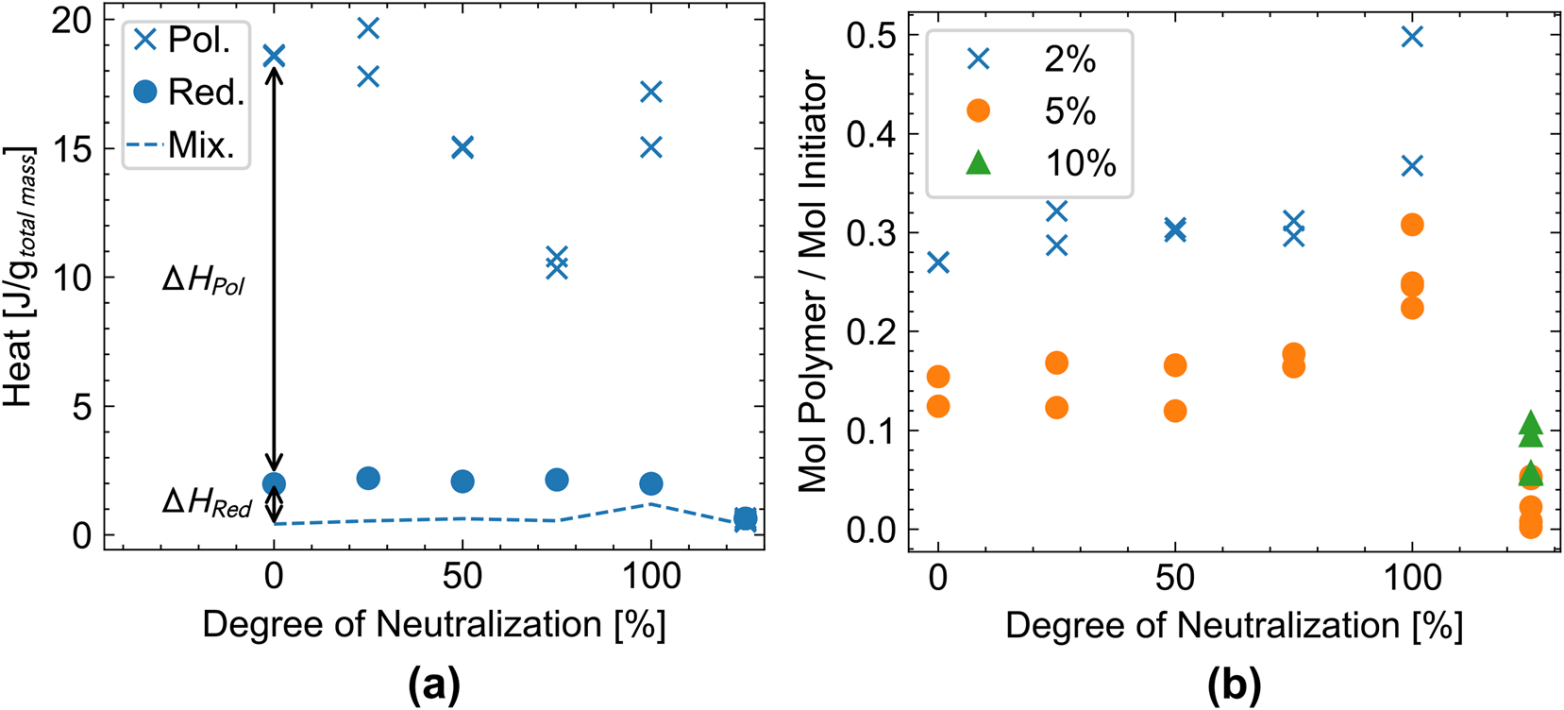
(a) The heat of the polymerization, of the redox reaction (which is determined by replacing acrylic acid with propionic acid), and the mixing (acrylic acid exchanged with propionic acid and peroxide exchanged with water) for an initiator dosage of 2 mol%. (b) Initiator efficiency is measured as the molar amount of polymer per theoretically produced radical by the initiator.

The redox-initiated polymerization of acrylic acid at different pH levels is conducted using the TBHP/SFS/Fe/EDTA initiator system described above; the pH is adjusted with NaOH, and the acrylic acid solid content is kept constant at 2%. The experimentally measured heat release is plotted as a function of the neutralization degree in Fig. 4a. To obtain the heat of polymerization from the total experimental heat, we subtract the heat of the redox system, as shown with a pair of arrows, for the neutralization degree of 2%. The heat of the redox reaction is determined by replacing acrylic acid with propionic acid, while keeping all other parameters constant. The individual values are reported in Table 2. We note that higher heats at the same neutralization degree usually correlate with higher yields (see Table S2).

**Table 2:** The mean heat of the polymerization (*Q̄*_Pol_, of two measurements), the heat of the redox reaction (*Q*_Redox_, acrylic acid exchanged with propionic acid), and the mixing heat (*Q*_Mix_, acrylic acid exchanged with propionic acid and peroxide exchanged with water) for an initiator dosage of 2 mol%, normalized to the total sample mass of 4 g

Neutralization degree [%]	*Q̄*_Pol_ [J g^−1^]	*Q*_Redox_ [J g^−1^]	*Q*_Mix_ [J g^−1^]
0	18.6	2.0	0.4
25	18.7	2.2	0.6
50	15.1	2.1	0.6
75	10.6	2.2	0.6
100	16.1	2.0	1
125	0.6	0.7	0.5

The mixing heat (*i.e.*, the heat obtained by mixing propionic acid and peroxide with water and therefore assuring the exclusion of a polymerization and a redox reaction) is 0.5 J g^−1^ to 1.0 J g^−1^ (normalized to total mass, see Table 2) and therefore very low compared to the reaction heat of the redox and polymerization reaction.

The heat released by the redox process (using propionic instead of acrylic acid to suppress polymerization) shows minimal variation up to a neutralization degree of 100%. At values above 100% neutralization, *i.e.*, at high pH, the heat release from the redox-initiator system declines sharply. This could be due to the partial precipitation of iron hydroxide in a basic environment, which hinders the catalysis of the redox reaction. Additionally, the generation of the initiator radical (eqn (3a)) involves the formation of a hydroxide ion. Under basic conditions, the reaction equilibrium is therefore shifted towards the educts. In the catalytic cycle, the generated Fe(iii) from eqn (3a) is regenerated by reduction with sodium hydroxymethanesulfinate (eqn (3b)).

Since the redox initiator system does not work in a basic environment, the discussion of the conversion is split into two sections: one for acidic environments and one for basic environments.

3a ROOH + Fe^2+^ → RO˙ + OH^−^ + Fe^3+^

3b 2 Fe^3+^ + NaSO_2_CH_2_OH + 2 OH^−^ → 2 Fe^2+^ + NaSO_3_CH_2_OH + H_2_O

The change in reactivity of the redox system can also be seen in Fig. 4b. The molar amount of polymer (calculated from the molar amount of reacted acrylic acid divided by the molar mass of the polymer) is divided by the molar amount of initiator. This estimates the initiator's efficiency, which is relatively low but within a reasonable range,^36^ probably due to the high dilution of the system and the presence of oxygen (no degassing step). The initiator efficiency remains relatively stable up to 75 percent neutralization (Fig. 4b), then increases to 100% and sharply drops to 125% as the initiator system exhibits negligible reactivity in the basic environment. We additionally note the significant difference in initiator efficiency between the two mol% initiator dosage (blue crosses in Fig. 4b) and the five mol% dosage (orange circles in Fig. 4b). It appears that the increase in initiator from 2% to 5% does not lead to a significant increase in polymer chains, resulting in a significantly lower initiator efficiency for the larger initiator dosage. Most likely, this can be explained by increased radical recombination.

### 3.3 Conversion of acrylic acid

#### 3.3.1 Conversion at low pH

**Fig. 5:**
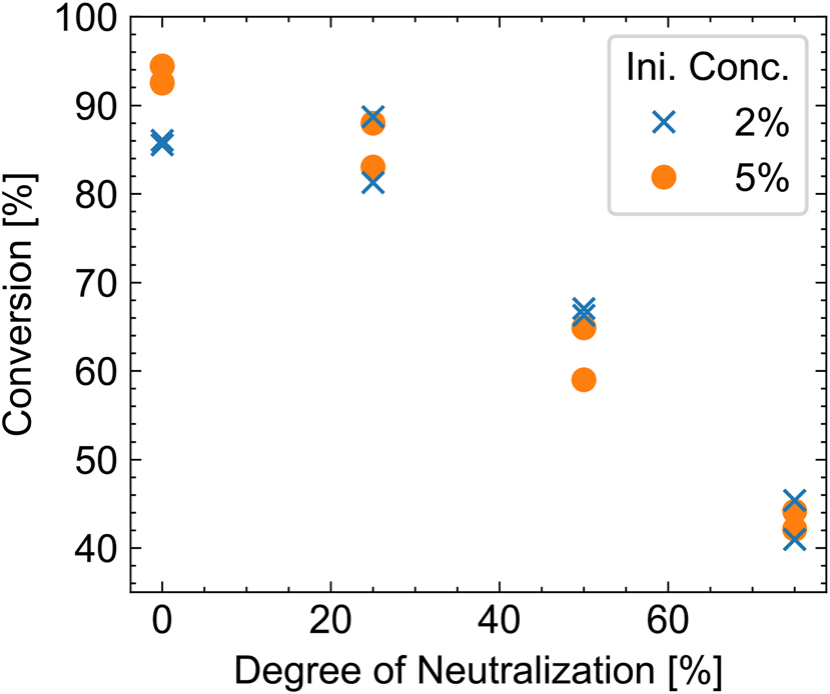
Conversion determined by HPLC. The different colors represent different initiator amounts (2 and 5 mol%).

We measure the conversion of acrylic acid after polymerization by HPLC at different degrees of neutralization (Fig. 5). With increasing amounts of sodium hydroxide, the conversion decreases from over 90% in the absence of any base to below 50% conversion with 75% degree of neutralization. The constant heat release of the redox reaction indicates that a similar quantity of radicals is generated (Fig. 4a and Table S4). Therefore, the difference in conversion is due to changes in the polymerization kinetics. Similar results were obtained both experimentally and through simulation.^2,5,7^ With increasing amounts of sodium hydroxide, more monomers are ionized. These ionized monomers will experience repulsive coulombic forces with the growing polymer chain (which is also anionically charged), leading to a lower local monomer concentration at the growing polymer and a decreasing polymerization rate. This reduction in the progression rate leads to radical depletion through recombination, thereby halting the reaction at low yields.

#### 3.3.2 Conversion at high pH

An acrylic acid neutralization degree of 100% leads to pH values of 8 and larger (see Table S2). This causes at least partial precipitation of the iron as iron hydroxide, rendering the redox system unreliable (see Section 3.2).

**Fig. 6:**
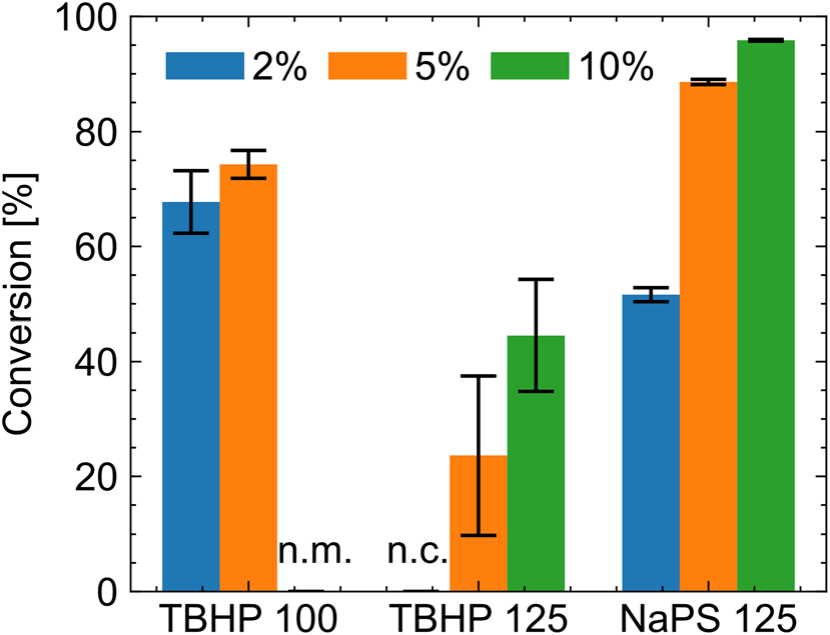
Conversion for different neutralization degrees and initiator systems. TBHP is the redox system containing *tert*-butyl hydroperoxide, and NaPS is sodium persulfate; the number behind the redox system is the degree of neutralization. Blue depicts polymerization with 2% initiator, orange 5% initiator and green 10% initiator. The error bar is the standard deviation. TBHP 100 10% was not measured (n.m.), and TBHP 125 2% has no conversion (n.c.).

We focus on the conversion dependence on the pH. Interestingly, a high conversion of up to 93% (see Table S2) can be achieved at full neutralization (pH 8.3). The redox system remains active at this pH, and all monomers are ionized, leading to Coulomb repulsion. Accordingly, we expect the radical termination rate to decrease, leading to a higher conversion rate because the radicals are depleted more slowly. The situation changes once more sodium hydroxide is added, and the pH is over 10. Now, the *tert*-butyl hydroperoxide-based redox system no longer works (the measured redox reaction heat is significantly reduced), resulting in low yields. Only a change of the initiator system to sodium persulfate (see Table 1) obtained good conversions at high pH values, but this system is only effective at high pH.^37^ Sodium persulfate was only used for this section (Fig. 6).

### 3.4 pH-dependent molar mass variation

**Fig. 7:**
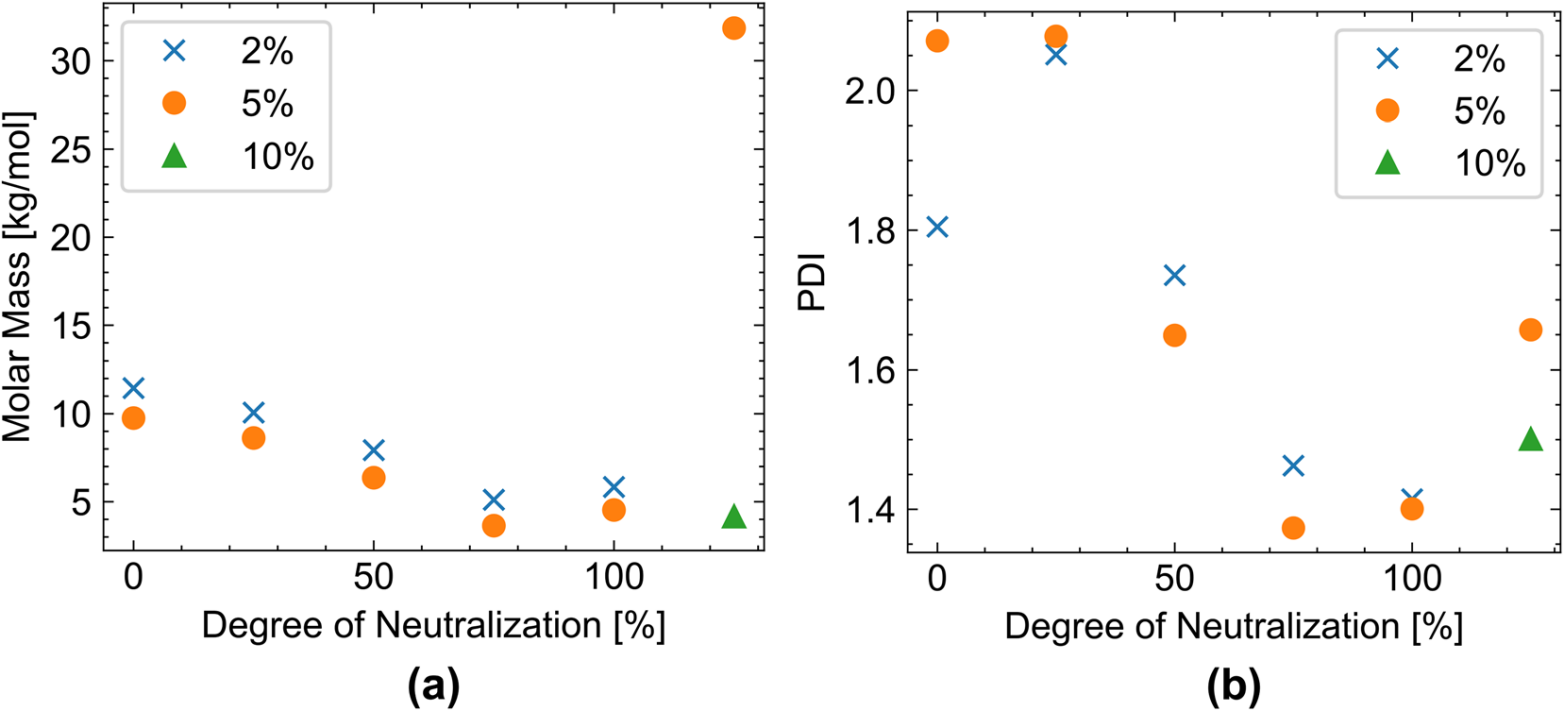
Variation of (a) molar mass and (b) PDI (polydispersity index) with increasing addition of sodium hydroxide (degree of neutralization). Blue depicts polymerization with 2% initiator, orange 5% initiator, and green 10% initiator; mean values of at least two measurements are shown. Individual values can be found in Table S2.

The molar masses of the obtained polymers are shown in Fig. 7 and Table S2. Up to 75 mol% sodium hydroxide addition, the molar mass of the obtained polyacrylic acid decreased, which coincides with the decrease in conversion in this range (Fig. 5a). A larger amount of initiator decreases molar mass only slightly, which again confirms the low initiator efficiency at higher dosages (Fig. 4b).

At 100% neutralization, the molar mass increases slightly to 5.84 kg mol^−1^ and 4.53 kg mol^−1^ for 2% and 5% initiator respectively compared to 5.11 kg mol^−1^ and 3.64 kg mol^−1^ for 2% and 5% initiator respectively at 75% neutralization, which corresponds to the increase in conversion (Fig. 7). At 125% sodium hydroxide addition, molar mass and PDI increase again (Table S2). This is likely due to the low reactivity of the redox system (TBHP/SFS/Fe). Fewer radicals are generated, and fewer polymer chains are initiated, resulting in a higher molecular mass. Additionally, no difference in molar mass or PDI is observed with respect to initiator concentration, further highlighting that the increase in initiator concentration primarily leads to more radical recombination rather than to an increase in polymer chains.

### 3.5 Polymerization heat at different pH values

**Fig. 8:**
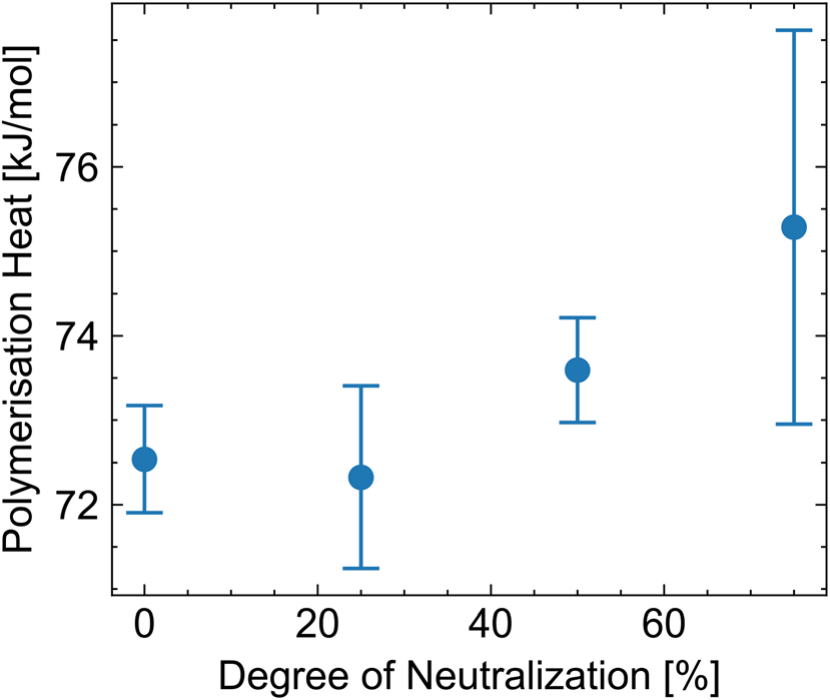
Polymerization heat for different neutralization degrees.

The polymerization heat can be calculated from the measured heat and the conversion (measured by HPLC) (Fig. 8). We measure a polymerization heat of (72.5 ± 0.6) kJ mol^−1^ for acrylic acid in water at a neutralization degree of 0%. The polymerization heat at 25% neutralization is very similar at (72.3 ± 0.3) kJ mol^−1^. However, at higher degrees of neutralization, the heat increases to up to (75.3 ± 2.3) kJ mol^−1^ for 75% neutralization. For neutralization degrees of 100% or higher, polymerization heat cannot be measured because radical initiation is inactive under basic conditions. Below this threshold, the dependence of the polymerization heat on the neutralization degree is hypothesized to arise from different pH-dependent ionization of the monomer and polymer. Increasing the polymer charge density alters polymer–solvent interactions and induces electrostatic chain stretching, leading to conformational changes reflected in the measured polymerization enthalpy.^38,39^

Such observations have been described for a solvent change. Gromov *et al.* report a polymerization heat of 77.4 kJ mol^−1^ for the polymerization of acrylic acid in water and 59.0 kJ mol^−1^ in dimethyl sulfoxide.^8^ Gromov assumes a similar interaction of the monomer (acrylic acid) with the solvent water and dimethyl sulfoxide, but a significantly different interaction of the polymer (polyacrylic acid). Water is a better solvent for polyacrylic acid, and the chain conformation will be different from that in dimethyl sulfoxide. Similarly, an increase of the polymerization heat from 41.8 kJ mol^−1^ to 60.7 kJ mol^−1^ through the dilution of methacrylic acid with methanol is observed.^15^ Joshi *et al.* explain this increase mainly as due to the loss of intermolecular association in the monomeric state upon diluting the methacrylic acid with methanol.^15^

In general, all polymerization heats reported here are higher than the 67 kJ mol^−1^ for pure acrylic acid,^15,17^ but lower than literature values with water as a solvent. Evans *et al.* report 77.5 kJ mol^−1^, McCurdyet al. 77.0 kJ mol^−1^ and Gromov *et al.* 77.4 kJ mol^−1^.^8,16,18^ The difference between our values and the literature values might be due to the high dilution in our system, which affects the polymerization heat, as described by Joshi (see above).^15^

We attribute the change in polymerization heat to changes in the polymer's conformation and solubility. There are many more polymers that likely exhibit pH-sensitive polymerization heat, but this topic is not well researched. There are several reactive groups often contained in polymers that are pH-sensitive, such as carboxylic acids, sulfonic acids, phosphoric acids, amino acids, boronic acids, or amides.^40^ In this paper, the change in polymerization heat was demonstrated for acrylic acid, a polymer containing a carboxylic acid group.

### 3.6 Determination of the polymerization rate from calorimetry data

#### 3.6.1 Time correction of the calorimetry data

**Fig. 9:**
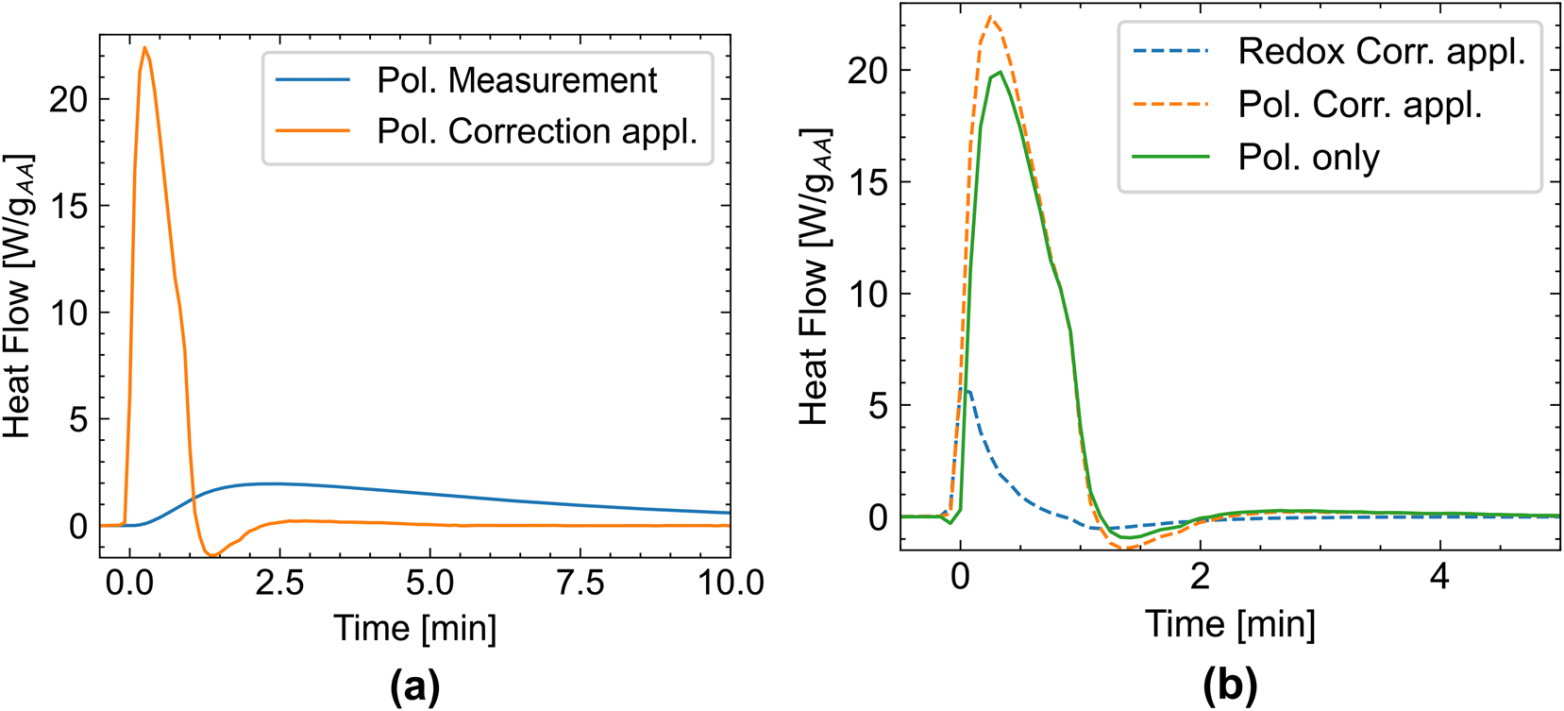
Polymerization at 0% degree of neutralization and a 2% initiator dosage. (a) Original heat flow data from the measurement (blue) and time-corrected heat flow data from applying eqn (2). (b) Time-corrected heat flow data from the polymerization measurement (dotted orange), the redox measurement (acrylic acid exchanged with propionic acid, dotted blue), and the polymerization-only data obtained by subtracting the redox heat flow data from the polymerization heat flow data. More curves are shown in Fig. S6.

To determine the polymerization rate from the calorimetry data, we need to precisely measure the relationship between heat development and time. Therefore, it is necessary to correct for the isothermal calorimeter's thermal lag. We use eqn (2) for this correction.^23^Fig. 9a depicts the effect of this mathematical correction. The measured signal (blue curve) becomes significantly shorter and exhibits an increased maximum heat flow (orange curve). A small overcorrection could not be avoided in our data treatment, as evidenced by the negative heat flow after polymerization, which is an artifact of the correction. In the next step, heat flow not originating from the polymerization was corrected for by subtracting the redox measurement (obtained from a measurement of the same system but replacing acrylic acid was exchanged with propionic acid) from the polymerization measurement, resulting in the green polymerization only curve in Fig. 9b, which only contains the polymerization heat and is time corrected.

The polymerization heat can be converted to monomer conversion using the measured polymerization heat of 72.54 kJ mol^−1^. The polymerization rate was then obtained by dividing the heat flow at 5% conversion with the polymerization heat and the molar mass of acrylic acid. In the next step, the data were normalized to the initial monomer concentration of 0.278 mol L^−1^.

**Fig. 10:**
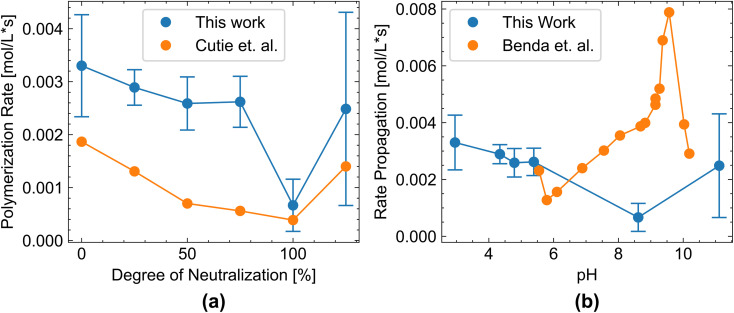
Polymerization rate in dependence of pH left. (a) Cutie, (b) Benda. Standard deviation as error bars. pH was measured after polymerization.

Fig. 10 compares the polymerization rate obtained in this work with data obtained with different techniques. Cutié *et al.*^7^ monitored the monomer conversion by NMR, and Benda *et al.*^6^ used a stirred rotary dilatometer. Although we observe a high standard deviation at high pH values, since the initiator system is at low reactivity, our data falls within a similar range and exhibits a similar pH dependence as Cutié *et al.* and various other works.^5,7,41,42^ First, the polymerization rate decreases with increasing pH as the acrylic acid monomer becomes increasingly deprotonated, leading to greater coulombic repulsion between the monomer and the growing polymer chain. This trend reaches its minimum at neutralization. After full neutralization, the polymerization rate increases again. This is explained by the formation of ion pairs between the growing chain and the monomer.^5,6^ Different reaction conditions can account for the difference between the values obtained in this paper and those reported by Cutié *et al.*, who demonstrated that temperature, monomer concentration, and initiator concentration affect the polymerization rate.^7^ This paper uses an acrylic acid concentration of 0.28 mol L^−1^ and a *tert*-butyl hydroperoxide concentration of 0.0056 mol L^−1^, whereas Cutié *et al.* used a an acrylic acid concentration of 2.8 mol L^−1^ and a sodium persulfate concentration of 0.0023 mol L^−1^. Especially a higher monomer concentration, closer to industrial conditions, would lead to higher polymerization rates and likely lead to an autoacceleration effect through a heat increase.

A more significant deviation occurs between our work and Benda *et al.* This is most likely because Benda *et al.* perform inverse emulsion polymerization and use ammonia as a neutralization agent at an acrylic acid concentration of 3.53 mol L^−1^ and an ammonium persulfate concentration of 0.0112 mol L^−1^. Still, Benda *et al.* also observe a trend of first decreasing and then increasing polymerization rates with pH.

Another key finding in our work is the importance of accurate calibration. The time correction is based on a calibration run where resistors produce a known heat signal. This heat signal should be as close as possible to the measured signal. The medium should be the same, and the resistor should be placed in the middle of the solution. Furthermore, the signal should be similar in evolved heat and the time the heat evolved. The data shown here are based on a 200 s heat pulse, similar to the duration of a polymerization reaction. If a heat pulse with 1 h is used, the calibration coefficients differ greatly ε* increases to 1.065 instead of 1.044 (for the 200 s heat pulse), *τ*_1_ is decreased to 305.21 s from 316.43 s, and *τ*_2_ is increased to 106.97 s from 57.91 s. These changes in the calibration result in a 60% increase in the polymerization rates (Fig. S3). We choose values for the 200 s heat pulse as it is a good balance between a short pulse which is similar to the polymerization signal and a long pulse which minimizes experimental errors due to timing issues (the heat signal was manually switched on an of with an approximate time error of 0.5 s). More details, including a sensitivity analysis of the calibration pulse and, in particular, of how uncertainties in ε*, *τ*_1_, and *τ*_2_ affect the propagation rates, are provided in the SI.

The time correction in an isothermal calorimeter is under development, and it is currently unclear which approach is best. In 2025, Lange *et al.* compared the approach presented in this paper, which uses a time-correction protocol adapted from Ejvu, with a deconvolution approach.^27^ Also in 2025, John *et al.*^29^ used a similar approach to Ejvu, introducing an additional term to improve accuracy. Both papers provide a more detailed account of the current challenges of time correction.

#### 3.6.2 Method comparison

Time-correction protocols for isothermal calorimetry are actively studied with different theoretical approaches^23,28^ and recent practical applications in cement chemistry.^27,29^ In the field of polymerization chemistry, a proper time correction of the heat flow data can simultaneously determine polymerization heat and the polymerization rate as shown above. Here, we briefly compare the time-corrected calorimetry data described above with existing methods to determine the polymerization heat or the rate.

The earliest determinations of the polymerization heat of acrylic acid employed setups with substantial thermal lag and coarse sampling. Evans *et al.*^16^ relied on a Dewar vessel with temperature readings at 30 s intervals, while Joshi *et al.*,^15^ using the Tong apparatus, achieved sampling intervals of about 60 s; in both cases, heat-transfer delays and long sampling times rendered kinetic analyses impossible. Subsequent isothermal calorimetric studies by McCurdy *et al.*^18^ and Gromov *et al.*^8^ did not specify acquisition rates but necessarily shared the intrinsic heat-transfer delay of such instruments, again compromising kinetic applicability without appropriate time corrections. Differential scanning calorimetry (DSC) offers intrinsically high temporal resolution, making it suitable for reaction-rate determination.^19^ However, the very small sample masses (1 mg to 10 mg) limit the accurate control of yield and conversion, which likely accounts for the lower reported polymerization heat compared to Joshi's value (Table 3).

**Table 3:** Previous studies on the polymerization heat of acrylic acid. IC: isothermal calorimeter, QA: quasi-adiabatic

Author	Year	Method	Data interval [s]	Δ*H*_*p*_ [kJ mol^−1^]
Evans^16^	1947	QA	30	77.5
Joshi *et al.*^15,43^	1962	Custom IC	60	67.0
McCurdy^18^	1964	IC	Unknown	77.0
Gromov^8^	1980	IC	Unknown	77.4
Kao^19^	2002	DSC	0.1	61.8
This study	2025	IC	0.5	72.5–75.3

On the other hand, methods commonly employed to determine the rate of polymerization are near-infrared spectroscopy,^2^ dilatometry,^5,6^ NMR,^7^ Raman spectroscopy,^9^ DSC^10^ or light scattering.^11^ However, these methods cannot, in principle, determine the enthalpy of polymerization. The only exemption is DSC measurements, which have proven unreliable for measuring polymerization heat, as discussed above, because the small sample size hinders precise determination of conversion.

Finally, we briefly compare the reaction calorimeter. A reaction calorimeter is used to monitor reaction kinetics under process-like conditions and to measure polymerization heat.^14^ Lamb *et al.* used such a system to measure the polymerization heat of styrene, resulting in an average polymerization heat of 62.8 kJ mol^−1^ with a standard deviation of 8.6 kJ mol^−1^. They attribute the large standard deviation to slow polymerization at low heat flow, which the calorimeter was unable to detect. Compared to a reaction calorimeter, the method described here is a significant improvement in sensitivity. The isothermal calorimeter in this study has a detection limit of 4 µW and a baseline drift of 5 µW over 24 h. This is enough to measure the heat of the redox system and even the heat generated by stirring water. Therefore, we can also measure slow polymerization processes with low heat flow, resulting in an average deviation of 0.9 kJ mol^−1^ for the polymerization heat. This accuracy is necessary to measure the small differences in polymerization heat resulting from changes in pH.

## 4 Conclusion

In summary, we successfully used time-corrected isothermal calorimetry to measure the heat of polymerization and the polymerization rate for acrylic acid at different degrees of neutralization. We measure a polymerization heat of (72.5 ± 0.6) kJ mol^−1^ up to (75.3 ± 2.3) kJ mol^−1^ for a highly diluted system (two mass percent acrylic acid), depending on the sodium hydroxide content. To our knowledge, the dependency of the polymerization heat of acrylic acid on pH has not been reported previously.

Furthermore, we measured the polymerization rate and found values close to those previously measured. The results show that the method provides simultaneous access to polymerization heat and kinetic parameters, streamlining the characterization of pH-sensitive polymers. In further studies, a further development of the initiator system would be an interesting objective. This could be achieved by using a photoinitiator and incorporating a lamp into the calorimeter cells.^44,45^ Another interesting objective is to increase the monomer concentration above 20%, for a better comparison with typical industrial conditions.

## Conflicts of interest

There are no conflicts to declare.

## Supplementary Material

RA-016-D5RA09826B-s001

## Data Availability

The data supporting this article have been included as part of the supplementary information (SI). Supplementary information is available. See DOI: https://doi.org/10.1039/d5ra09826b.
