# Supplementary material for: Isothermal heat flow calorimetry for one-step determination of polymerization heat and rate of poly(acrylic acid) at varying pH
Source: RSC Adv. 2026 Feb 18;16(11):9722–32. doi: 10.1039/d5ra09826b (PMC12915094; doi:10.1039/d5ra09826b)
Supplement: RA-016-D5RA09826B-s001 [file RA-016-D5RA09826B-s001.pdf]

Supplementary Information (SI) for RSC Advances.
This journal is © The Royal Society of Chemistry 2026

Supporting Information for: Isothermal Heat Flow Calorimetry for One-Step Determination of Polymerization Heat and Rate of Poly(acrylic acid) at Varying pH

Sebastian Remke[1], Gaurav Sant[2,3,], and Torben Gädt[1]

[1]Chair for the Chemistry of Construction Materials, TUM School of Natural Sciences, Technical University of Munich, Lichtenbergstraße 4, 85748 Garching, Germany

[2]Laboratory for the Chemistry of Construction Materials (LC²), Department of Civil and Environmental Engineering, University of California Los Angeles, Los Angeles, CA 90095, USA

[3]Institute for Carbon Management (ICM), University of California Los Angeles, Los Angeles, CA 90095, USA

1 Polymerization Conditions

1.1 Optimization of the redox initiator system for acrylic acid polymerization at room temperature

First, we determine the heat generated by the redox initiator system in the absence of acrylic acid, followed by a polymerization run with a solution of two mass percent of acrylic acid and 1 mol% initiator (TBHP) relative to the monomer acrylic acid (see Table **??**). Each composition was tested four times.

When only the TBHP/SFS redox pair was employed, an irregular induction period preceded the redox reaction, shifting the heat-flow maximum by 10 to 100 min while the total heat release (i.e., overall reaction degree; see Figure S1) remained unchanged. The variance in induction time, also reported by Lamb et al. and Kohut-Svelko et al. [1, 2], hindered the reliable determination of heat and initiation. The polymerization without iron(II) ions as a catalyst shows the same variable induction period of a few minutes and produces a significantly lower maximum heat flow (corresponding to the reaction rate) (see Figure S4). This results in a comparatively large PDI of 2.60 $\pm$ 0.09 and a molecular weight of 33.3 $\mathrm{kg\,mol^{-1}} \pm 4.3\,\mathrm{kg\,mol^{-1}}$.

This resulted in precisely reproducible reaction timing but produced significant variance in the total heat generated (conversion, Figure S1). The heat variance and, thus, different reaction rates could occur due to partial oxidation of the iron(II) ions by oxygen dissolved in the water or in the syringe. The variance in initial heat evolution could be resolved by combining peroxide, a reductant, and an iron(II) salt. The combination of TBHP/SFS/Fe reliably triggers polymerization upon mixing, i.e., without any induction time, resulting in a molar mass of 17.1 $\mathrm{kg\,mol^{-1}} \pm 1.9\,\mathrm{kg\,mol^{-1}}$, and a PDI of 1.67 $\pm$ 0.09.

1.2 Exemplary experiment description

For an experiment set with 2 % initiator, 50 % neutralization with the TBHP initiator system, the following runs were measured.

For a polymerization run 400 mg of a 20 % acrylic acid solution and 1824 mg water were put in the beaker of the calorimetry cell before adding 555 mg of a 1 M sodium hydroxide solution. One syringe was filled with 400 mg of a 0.5 % *tert*-Butyl hydroperoxide solution, the second syringe with 376 mg of a1 % sodium formaldehyde sulphoxylate solution and the third syringe with 444 mg of a 0.05 % $FeSO_4$ solution (with an equimolar amount of EDTA already in this solution).

For a redox reaction run 400 mg of a 20.5 % propionic acid solution (higher concentration to com-

pensate for the higher molar mass) and 1826 mg water were put in the beaker of the calorimetry cell before adding 555 mg of a 1 M sodium hydroxide solution. One syringe was filled with 400 mg of a 0.5 % *tert*-Butyl hydroperoxide solution, the second syringe with 376 mg of a1 % sodium formaldehyde sulphoxylate solution and the third syringe with 444 mg of a 0.05 % $FeSO_4$ solution (with an equimolar amount of EDTA already in this solution).

For a mixing heat run 400 mg of a 20.5 % propionic acid solution (higher concentration to compensate for the higher molar mass) and 1826 mg water were put in the beaker of the calorimetry cell before adding 555 mg of a 1 M sodium hydroxide solution. One syringe was filled with 400 mg water, the second syringe with 376 mg of a1 % sodium formaldehyde sulphoxylate solution and the third syringe with 444 mg of a 0.05 % $FeSO_4$ solution (with an equimolar amount of EDTA already in this solution).

2 investigation of the redox reaction

If only the redox pair TBHP/SFS was used, the redox reaction occurred after a somewhat irregular induction period resulting in a shift of the heat maximum by 10 to 100 min but with constant total heat (corresponds to reaction degree, see Figure S1). The variance in induction time, also reported by Lamb et al. and Kohut-Svelko et al. [1, 2], hindered the reliable determination of heat and initiation. The induction time variance could be very strongly reduced by adding iron(II)sulfate as a catalyst [2]. This resulted in a precisely reproducible reaction timing but generated a significant variance in the generated total heat (conversion, Figure S1). The heat variance and, thus, different reaction degrees could occur due to the partial oxidation of the iron(II) ions by oxygen dissolved in the water or the syringe. The addition of a complexing agent like ethylenediaminetetraacetic acid (**EDTA**) can stabilize iron(II) by complex formation, thereby stabilizing the iron(II) against oxidation. The combination of TBHP/SFS and iron(II)-EDTA solution resulted in a well-timed and heat-consistent redox reaction (Figure S4 a).

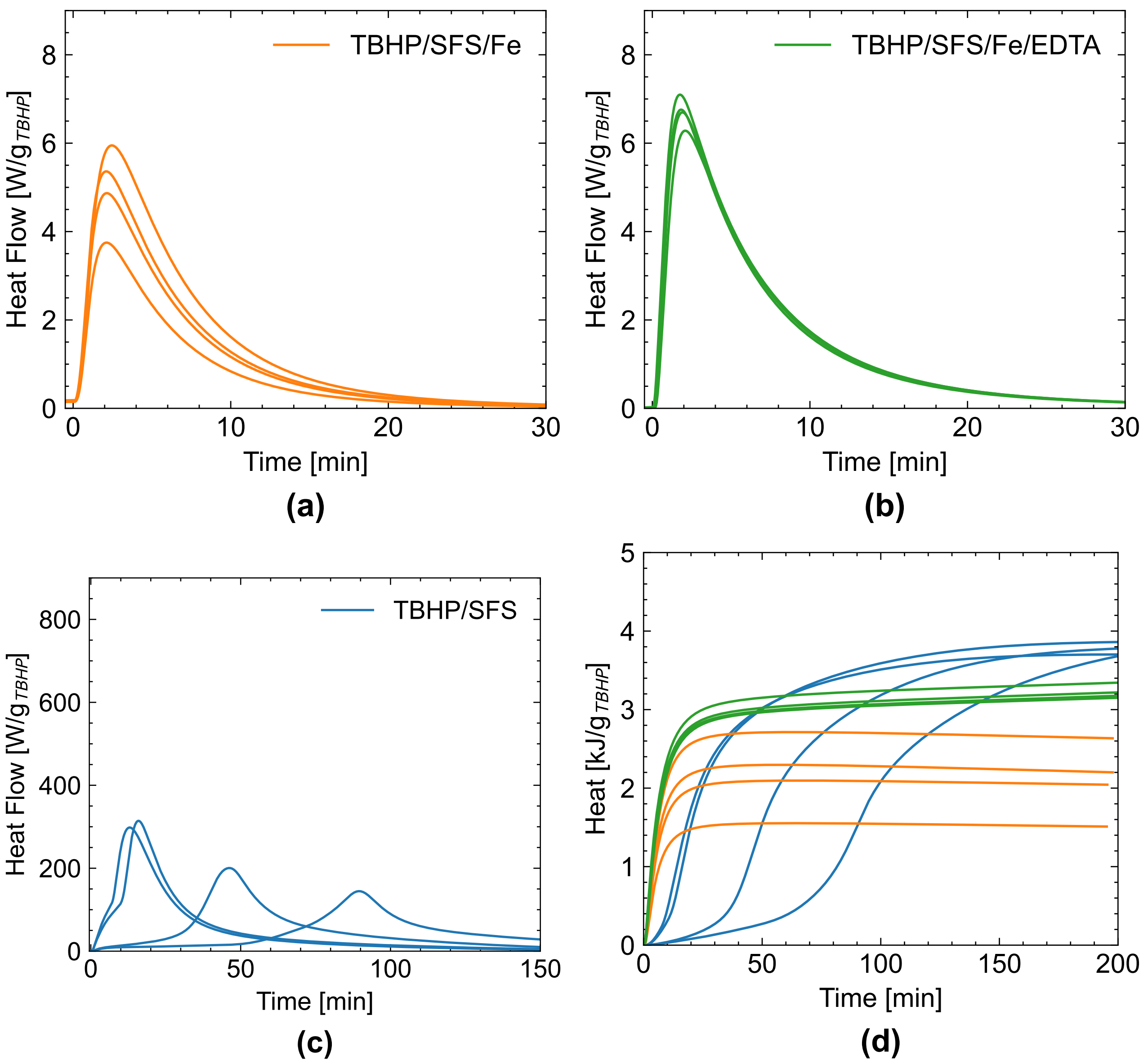

Figure S1: a) Heat flow for the redox pair TBHP/SFS,b) Heat flow for the redox pair TBHP/SFS with the addition of iron(II)sulfate as a catalyst, c) Heat flow for the redox pair TBHP/SFS with the addition of iron(II)sulfate as a catalyst and with ethylenediaminetetraacetic acid for stabilization, d) cumulative heat for the previous redox systems.

Statistical analysis of the heat correction

As described in the main article, after the calibration measurements, the individual correction factor ε^* is obtained by dividing the calculated heat by the resistor (Q_{calc}) and the measured heat (Q_{meas}). In the next step, equation 1 was used to fit the measured data (original) to the applied heat signal (pulse). This resulted in values for τ_1 and τ_2. These vary with pulse width; Table S1 provides an overview of how the calibration factors change with pulse width, ϵ epsilon is not shown because it is a device parameter that remained constant throughout the experiments.

$$P_{corr}(t) = \varepsilon^* \left(P_{raw}(t) + (\tau_1 + \tau_2)\frac{dP_{raw}(t)}{dt} + \tau_1\tau_2\frac{d^2P_{raw}(t)}{dt^2} \right) \tag{1}$$

For the measurements themselves, equation 1 is used to convert the raw thermal power ($P_{raw}(t)$) from the calorimeter measurement (ε is already included in the measurement) to the time corrected thermal power ($P_{corr}(t)$). Therefore, the accuracy of the corrected thermal power depends on the accuracy of ε^*, τ_1, and τ_2.

The polymerization rate (r_p) is obtained by dividing the thermal corrected thermal power ($\mathrm{W\,g^{-1}}$) at 5 % by the polymerization heat of acrylic acid ($1006.65\,\mathrm{J\,g^{-1}}$) before multiplying with the molar concentration of acrylic acid at reaction start ($0.2775\,\mathrm{mol\,L^{-1}}$) (equation 2). The polymerization rate is therefore directly calculated from the corrected thermal power and also depends on the accuracy of ε^*, τ_1, and τ_2.

To estimate how sensitive the polymerization rate is to a change in ε^*, τ_1, and τ_2, we changed one calibration factor (ε^*, τ_1, τ_2) at a time in the range of double the standard deviation calculated in Table S1 and calculated the corresponding polymerization rate (Figures S2). The slope of r_p versus ε^* and τ_1 (Figure S2 a and b) is comparatively small, indicating a modest effect of errors in ε^* and τ_1 on the polymerization rate. In contrast, the slope for r_p against τ_2 is significantly larger, which means that errors in τ_2 have a more significant influence than those for ε^* and τ_1. The values for the other calibration experiments are collected in Table S1. The most important difference here is between the τ_2 values. We show the r_p values obtained with the τ_2 value from the 60 minute calibration experiment in Figure S3. For our manuscript, we chose the 200 s pulse, as this time frame is close to the expected heat-up time for a polymerization event, and the obtained r_p values are closer to the literature values.

$$r_p = \frac{P_{corr}(t)}{1006.65\,\mathrm{J\,g^{-1}}}\,0.2775\,\mathrm{mol\,L^{-1}} \tag{2}$$

Figure S3 compares the literature value of Cutie et. al. [3] with the polymerization rates obtained from the 200 s pulse experiment and the 1 h pulse experiment. The longer calibration pulse results in approximately 60 % higher rate of polymerization, which is mainly caused by the larger τ_2 value.

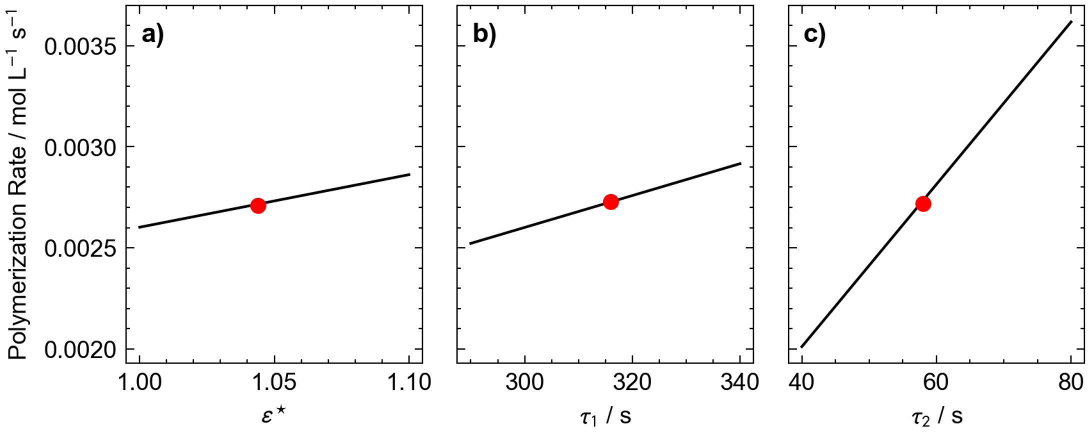

Figure S2: Change in the calculated rate of propagation for a variation of a) ε^* , b) τ_1, and τ_2 in the range of double the standard deviation calculated in Table S1, the other values are kept at the value originating from the 200 s pulse experiment. The red dot is the value obtained from the calibration run with a 200 s pulse (used in the publication).

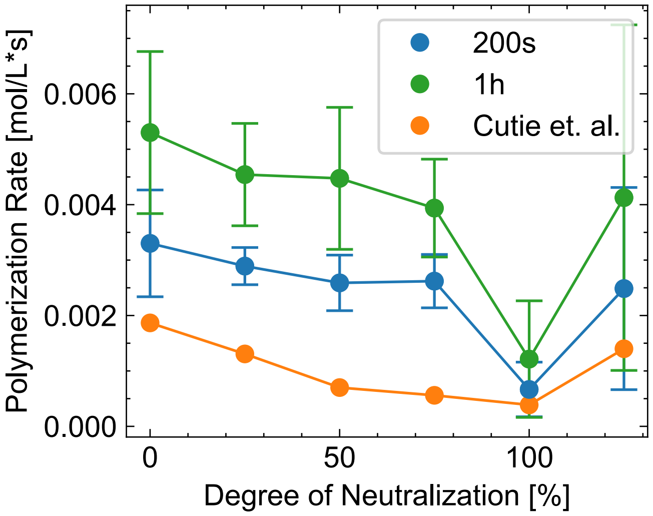

Figure S3: Comparison of the polymerization rate reported by Cutie et. al. [3] (orange), our data with a calibration pulse of 200 s (blue) resulting in a ϵ^* of 1.044, τ_1 of 316.43 and τ_2 of 57.91, and a calibration pulse of 1 h (orange) resulting in a ϵ^* of 1.065, τ_1 of 305.21 and τ_2 of 106.97.

Table S1: Values for the calibration factors ε^* , τ_1, τ_1 for different pulse widths (t; 100 s, 200 s, 1 h). With the corresponding expected value E and the standard deviation σ for these three calibration runs.

value	t: 100 s	t: 200 s	t: 1 h	E	σ
ε^*	1.046	1.044	1.065	1.052	0.011
τ_1	324.155 s	316.428 s	305.206 s	315.263 s	9.528 s
τ_2	62.347 s	57.913 s	106.967 s	75.742 s	27.132 s

3 Additional Figures

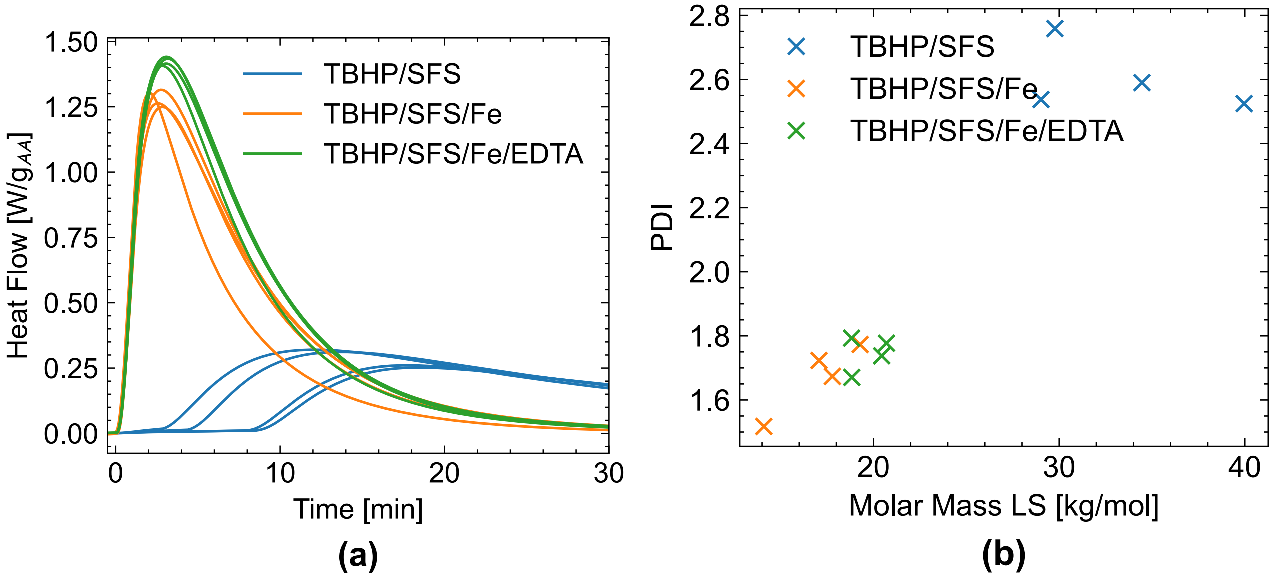

Figure S4: a) Heat flow for polymerization run using redox pair TBHP/SFS alone, with the addition of iron(II)sulfate as a catalyst (TBHP/SFS/Fe, and with ethylenediaminetetraacetic acid (TBHP/SFS/Fe/EDTA), b) associated molar mass and PDI to those polymerization runs.

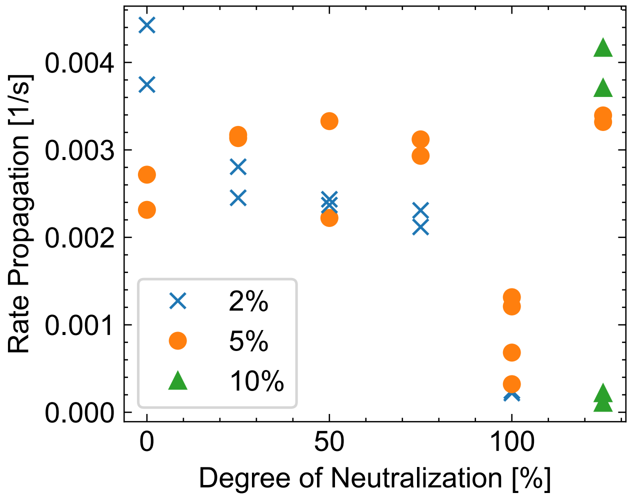

Figure S5: Polymerization rate in dependence of neutralization degree.

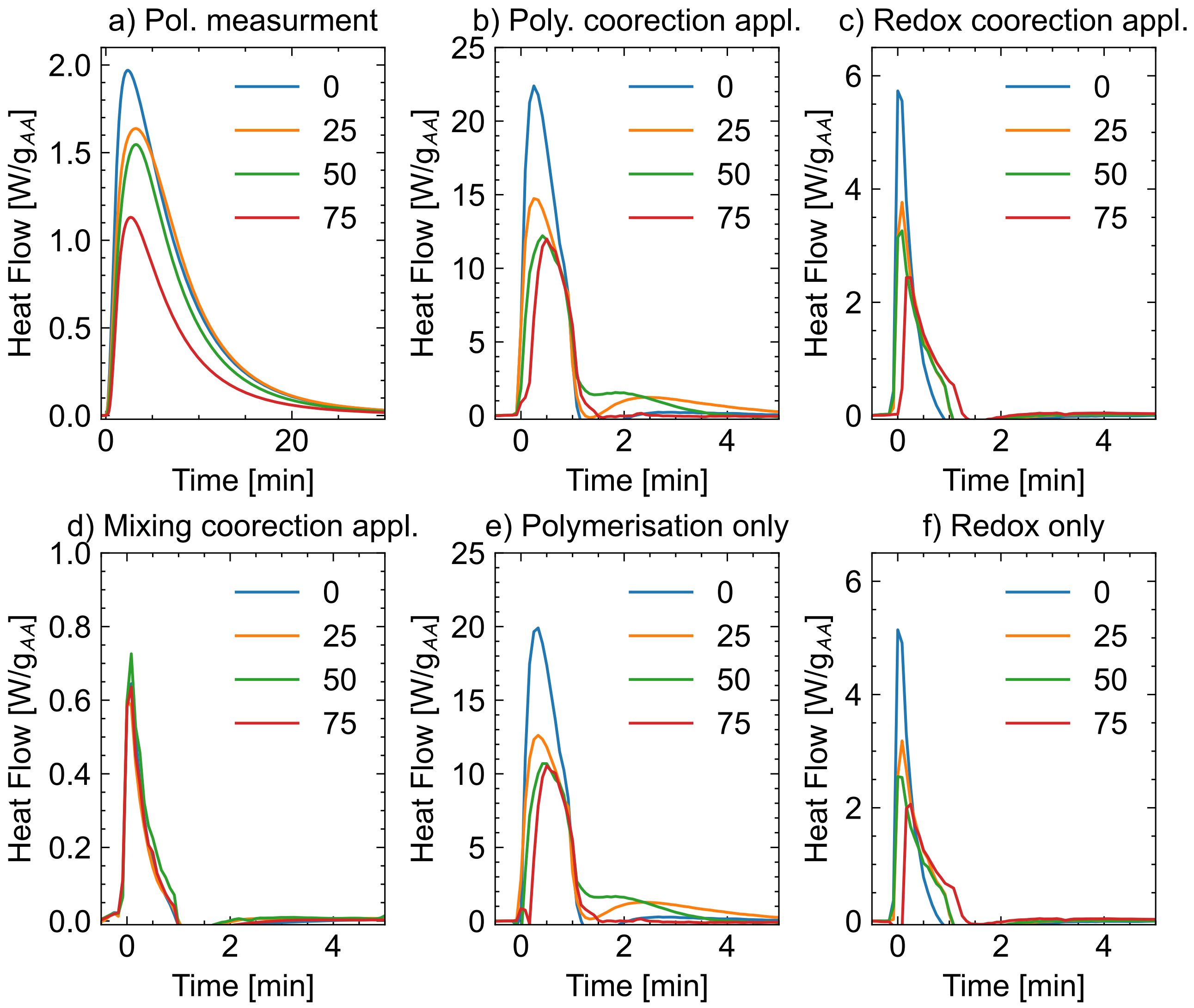

Figure S6: a) Measured polymerization run with the 2% of the TBHP/SFS/Fe/EDTA initiator system at different neutralization degrees (blue: 0%, yellow: 25%, green: 50%, red: 75%), b) time correction applied to the polymerization run, c) time correction applied to the redox run, d) Heat correction applied to the mixing run, e) result if c is subtracted from b (only polymerization heat), f) result if d is subtracted from c (only redox heat) c-d; all measurements were conducted with 2% initiator and normalized to the theoretical acrylic acid content (if propionic acid was used the mass of acrylic acid wich was exchanged was used for normalization).

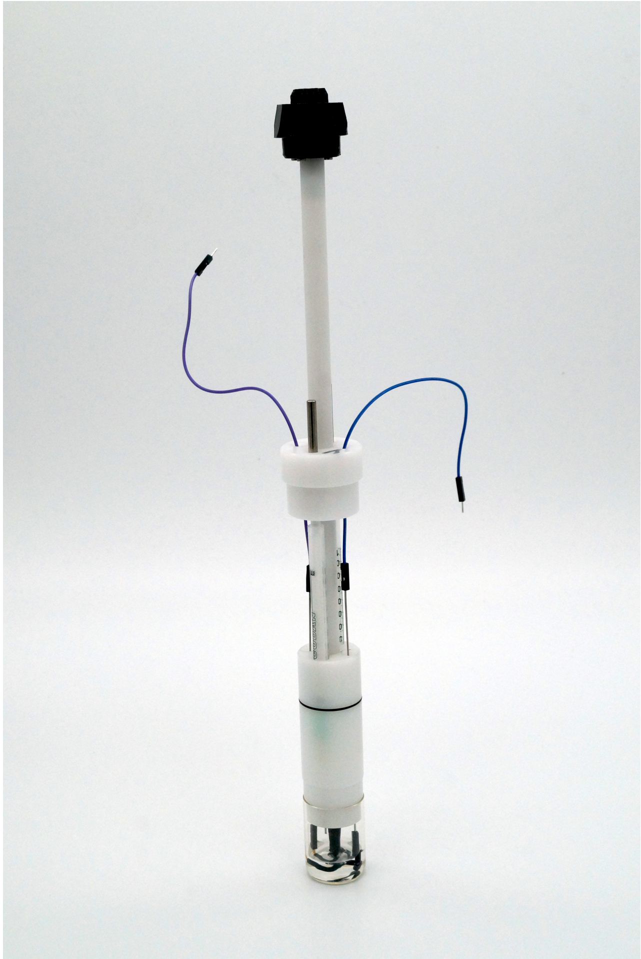

Figure S7: Picture of the In situ cell with resistor and cable for heat correction measurement attached. The plastic/graphite composite beaker was exchanged with a glass beaker for this picture.

4 Additional Tables

Table S2: Data obtained for the polymerization runs with the TBHP/SFS/Fe system. A decrease in pH with increasing initiator content can be observed, since the initiator was not taken into account for the neutralization degree. If no value for Rp_{Ini} is given 5 % conversion was not reached in the observed time frame.

NaOH $[\%\frac{mol}{AA}]^a$	pH	Ini $[\%\frac{mol}{AA}]^a$	Conv. [%]	Q_{meas} [J]	ΔH [kJ mol^{-1}]	M_{LS}^{b} [g mol^{-1}]	PDI	Rp_{Ini}^{c} [s^{-1}]	Rp_{Max}^{d} [s^{-1}]
0	2.8	2	86	74.5	72.8	11503	1.77	0.0160	0.0200
0	2.8	2	86	74.3	72.9	11407	1.84	0.0135	0.0194
0	3.2	5	94	88.8	73.0	8802	2.06	0.0083	0.0140
0	3.1	5	93	85.9	71.4	10703	2.08	0.0098	0.0132
25	4.3	2	81	71.1	72.1	10194	1.87	0.0101	0.0127
25	4.4	2	89	78.7	74.1	9934	2.24	0.0088	0.0101
25	4.4	5	88	83.0	72.0	7533	2.17	0.0114	0.0116
25	4.3	5	83	78.3	71.1	9710	1.99	0.0113	0.0115
50	4.8	2	67	60.3	73.0	7926	1.68	0.0085	0.0108
50	4.8	2	66	60.1	73.4	7933	1.80	0.0088	0.0112
50	4.8	5	65	65.5	73.4	5637	1.61	0.0080	0.0120
50	4.8	5	59	61.7	74.6	7092	1.69	0.0120	0.0132
75	5.3	2	41	41.3	75.2	4982	1.43	0.0083	0.0105
75	5.3	2	45	43.2	71.7	5241	1.49	0.0076	0.0098
75	5.4	5	42	49.0	76.0	3688	1.38	0.0106	0.0144
75	5.5	5	44	51.7	78.2	3595	1.37	0.0112	0.0149
100	8.7	2	73	68.8	78.2	7171	1.45	0.0008	0.0009
100	8.8	2	62	60.2	78.9	4506	1.38	0.0009	0.0010
100	8.7	5	93	88.9	76.3	5999	1.48	0.0047	0.0059
100	8.3	5	71	42.1	37.8	4182	1.37	0.0044	0.0050
100	8.6	5	77	60.2	56.8	4484	1.42	0.0012	0.0014
100	8.6	5	74	59.7	58.8	3462	1.33	0.0025	0.0027
125	10.1	5	18	25.8	111.2	31716	1.61	0.0120	0.0120
125	10.3	5	16	26.2	128.8	31990	1.55	0.0122	0.0122
125	12.3	5	29	6.0	4.6	18705	1.73	-	0.0004
125	12.2	5	45	12.0	15.7	12514	1.61	-	0.0004
125	10.5	5	41	9.1	10.3	11066	1.56	-	0.0001
125	11.6	5	9	8.9	26.4	59403	1.83	-	0.0002
125	11.6	5	8	9.1	23.8	57572	1.71	-	0.0000
125	9.4	10	33	60.9	70.2	4291	1.53	0.0134	0.0134
125	9.5	10	37	61.6	65.4	4597	1.88	0.0150	0.0152
125	9.5	10	55	80.9	76.5	3683	1.30	0.0004	0.0006
125	9.4	10	53	79.3	76.3	4038	1.30	0.0008	0.0009

[a] mol percentage to acrylic acid, [b] molar mass determined by light scattering detector, [c] rate of propagation at 5 % conversion, [d] maximum rate of propagation

Table S3: Data obtained for the polymerization runs with the NapS system

NaOH $\left[\%\frac{mol}{AA}\right]$[a]	pH	Ini $\left[\%\frac{mol}{AA}\right]$[a]	Conv. [%]	Q_{meas} [J]	ΔH [kJ mol^{-1}]	M_{LS}[b] [g mol^{-1}]	PDI
125	13.3	2	50	1.1	-1.3	9019	1.76
125	11.6	2	53	1.3	-1.0	8411	1.73
125	9.4	5	88	1.7	0.2	7786	1.77
125	9.5	5	89	1.6	0.1	7804	1.77
125	8.5	10	96	89.0	84.7	10677	1.81
125	8.9	10	96	91.8	87.2	9526	1.74

[a] mol percentage to acrylic acid, [b] molar mass determined by light scattering detector

Table S4: Redox and mixing heat for the TBHP/SFS/Fe system.

NaOH $\left[\%\frac{mol}{AA}\right]$[a]	Ini $\left[\%\frac{mol}{AA}\right]$[a]	Q_{Redox} [J]	Q_{Mixing} [J]
0	2	7.9	1.7
0	5	15.6	0.9
25	2	8.8	2.2
25	5	15.6	0.8
50	2	8.3	2.5
50	5	14.9	1.3
75	2	8.6	2.2
75	5	14.9	2.1
100	2	8.0	4.8
100	5	13.4	5.7
125	2	2.5	1.6
125	5	4.6	2.0
125	5	6.4	2.0
125	5	7.1	2.0
125	5	4.6	2.0
125	5	4.6	2.0
125	10	36.1	7.5

[a] mol percentage to acrylic acid
